# Ontario Veterinary College—New York State Veterinary College

**Published:** 1901-06

**Authors:** W. E. Howe

**Affiliations:** Graduate of New York State Veterinary College of ’97; U. S. Department of Agriculture, Bureau of Animal Industry, Local office, 1320 Stout Street, Denver, Col.


### CORRESPONDENCE.

### ONTARIO VETERINARY COLLEGE—NEW YORK STATE VETERINARY COLLEGE.

U. S. Department of Agriculture,
Bureau of Animal Industry,
Local office, 1320 Stout Street, Denver, Col.

Editor Journal of Comparative Medicine :

Sir : As a graduate of the Ontario Veterinary College and also of the New York State Veterinary College, I wish to correct some erroneous impressions left by J. P. Foster in his article in the March Journal of Comparative Medicine and Veterinary Archives. I want to give the Ontario Veterinary College all the credit to which it is entitled, and also wish the New York State Veterinary College to have what credit belongs to it.

If J. P. Foster would compare the two schools as they should be compared, from faculty to equipment and the work actually given to the student, he could come to no other conclusion than that if the student of three years at the New York State Veterinary College could not take the prizes from a man who had been two years at the Ontario Veterinary College, and then taken his third year at the New York State Veterinary College, it was simply because his mental calibre was deficient. When I say compare I do not mean a comparison simply of catalogues, but of the actual work required of the student.

When Dr. Foster attempts to state facts about different colleges he would do well to tell not only the truth but the whole truth. He refers to Toronto men taking prizes at the New York State Veterinary College in the years 1896 to 1897 and 1897 to 1898. I would like to ask who would take them if it were not a man from some other college, as 1897 was the first year the New York State Veterinary College had its doors open to students and had none of its men to compete with students from other colleges. The student who took the prize in 1898 was a student in my class at Toronto, and was a prize man there, if I remember correctly; but the prize won at the New York State Veterinary College was gained under similar circumstances as those won by H. R. Ryder and W. E. Howe in 1897.

We must give Toronto credit for being equal to any school of two years of six months each, but it cannot compare with one of three years of nine months each and the equipment of the New York State Veterinary College.

Dr. Foster refers to a comparison of the catalogues of the colleges' as if red ink and embossing had anything to do with the advantages of a college. Compare the catalogues as to their truthfulness, to see which one fulfils the promises and conditions as stated, and the New York State Veterinary College will lose nothing, nor will it detract from its neat and tasty appearance.

I believe the Ontario Veterinary College states that the students have the advantage of the college practice, but it is only a few of the favorites that ever see any practical work there, so what does the statement amount to in the catalogue? Other instances of catalogue promises not being fulfilled could be cited, but it is no use.

If Dr. Foster will search for the truth, the whole truth, and nothing but the truth, he will have no excuse for complaint against the New York State Veterinary College.

Respectfully, W. E. Howe,

Graduate of New York State Veterinary College of '97.

